# Authors’ Reply: Pyridine nucleotide regulation of hepatic endoplasmic reticulum calcium uptake

**DOI:** 10.14814/phy2.14258

**Published:** 2019-10-09

**Authors:** Kenneth L. McCormick

**Affiliations:** ^1^ University of Alabama Birmingham Birmingham Alabama

To echo your concern, we were cognizant that commercially available NADP was conceivably “contaminated” with a trace‐ but nevertheless germane‐ amount of NAADP. And, indeed, Sigma does not discount this possibility in their NADP compound (personal communication). If this contaminant was present, the attenuation of net ER calcium ascribed to NADP could be specious, that is, the uptake reduction due chiefly to an opening of a NAADP‐activated efflux channel in the endoplasmic reticulum(ER).

Moreover, we were mindful of the parabola‐shaped, dose–response effect of the ligand NAADP on its cognate channel. Accordingly, we painstakingly explored logarithmically different concentrations of NAADP alone (no NADP added), ranging from 0.5 nmol/L to 1 mmol/L, on both ER calcium uptake and egress. There was no effect of NAADP save a mild reduction of uptake at 1 mmol/L. As for this latter observation, at present there is no explanation.

Furthermore, In the Discussion section, arguments were adduced that our microsome preparation would doubtfully contain the enzymatic machinery or chemical conditions to generate NAADP from added NADP. Parenthetically, our microsomes are devoid of cytochrome c oxidase and monoamine oxidase (mitochondrial markers), and rich in glucose dehydrogenase, glucose 6 phosphatase, and NADPH cytochrome c reductase (all endoplasmic reticulum markers). Cytosolic, membrane, and nuclear enzymes are absent‐ this is significant insofar as the NAADP synthesizing enzyme CD38, an ADP‐ribosyl cyclase, has both transmembrane and nuclear envelope locations (Keng et al. 2000; Zhao et al. 2011).

As for the discordance in the IC_50_ values (≈0.5 mmol/L) for the inhibition of ER calcium uptake compared to Ca^++^ ATPase, bear in mind that the assay milieus are altogether different (namely, the pH, ionic strength, and buffer type), and especially the final concentration of free calcium (100 vs. 20 *µ*mol/L respectively). These physiochemical factors are relevant. As often with in vitro measurements, standard assays for Ca^++^ATPase or calcium uptake do not exist, and the assay variations are legend (Rej and Vanderlinde 1975; Webster et al. 1980; Erickson et al. 1987; Lund and Wiggins 1987; Yamamoto and Suzuki 1987; Missiaen et al. 1989; Hethey et al. 2002; Luo et al. 2010). In our experience, even the buffer type (MOPS, HEPES, TRIS, Imidazole) can be impactful, particularly in calcium uptake studies. Hence, comparisons between IC_50_ values concerning two different biochemical entities (uptake vis‐à‐vis ATPase), albeit related, and under disparate assay conditions, are unsurprisingly inexact.
